# Interleukin 6: at the interface of human health and disease

**DOI:** 10.3389/fimmu.2023.1255533

**Published:** 2023-09-28

**Authors:** Elena Grebenciucova, Stephen VanHaerents

**Affiliations:** Feinberg School of Medicine, Department of Neurology, Northwestern University, Chicago, IL, United States

**Keywords:** interleukin 6, IL-6, cytokine, neuro-inflammation, NPSLE, NMOSD, tocilizumab, sartralizumab

## Abstract

Interleukin 6 (IL-6) is a pleiotropic cytokine executing a diverse number of functions, ranging from its effects on acute phase reactant pathways, B and T lymphocytes, blood brain barrier permeability, synovial inflammation, hematopoiesis, and embryonic development. This cytokine empowers the transition between innate and adaptive immune responses and helps recruit macrophages and lymphocytes to the sites of injury or infection. Given that IL-6 is involved both in the immune homeostasis and pathogenesis of several autoimmune diseases, research into therapeutic modulation of IL-6 axis resulted in the approval of a number of effective treatments for several autoimmune disorders like neuromyelitis optica spectrum disorder (NMOSD), rheumatoid arthritis, juvenile idiopathic arthritis, polyarticular juvenile idiopathic arthritis, giant cell arteritis (GCA), and cytokine release syndrome, associated with SARS-CoV2 pneumonia. This review discusses downstream inflammatory pathways of IL-6 expression and therapeutic applications of IL-6 blockade, currently investigated for the treatment of several other autoimmune conditions such as autoimmune encephalitis, autoimmune epilepsy, as well as myelin oligodendrocyte glycoprotein associated demyelination (MOGAD). This review further highlights the need for clinical trials to evaluate IL-6 blockade in disorders such neuropsychiatric lupus erythematosus (SLE), sarcoidosis and Behcet’s.

## 1 Introduction

IL-6 was first described in 1973 as a protein secreted by T lymphocytes that aided B cell differentiation into antibody producing cells; thus, it first became known as ‘B cell stimulatory factor 2 (BSF2)’ (1). A decade later, other proteins previously known as hepatocyte stimulating factor, IFN-β2, as well as plasmacytoma growth factor were cloned and found to be identical to IL-6, first illustrating its pleotropic functionality. In 1988 at a conference titled ‘Regulation of the Acute Phase and Immune Responses: A New Cytokine,’ BSF2 was re-named into interleukin 6 (2). IL-6 is a small polypeptide (molecular weight of 19–28 kDa), comprised of four α helices. Usually existing in a monomer form, it consists of 184 amino acid residues, glycosylation sites and two disulfide bonds. IL-6 encoding gene is located on chromosome 7p and includes 4 introns and 5 exons (3). It is produced by B lymphocytes, T lymphocytes, macrophages, including microglia, as well as fibroblasts, keratinocytes, mesangial cells, vascular endothelial cells, mast cells, and dendritic cells. IL-6 expression is mainly activated by interleukin 1 β (IL-1β) and tumor necrosis factor-alpha (TNFα); however, there are also other ways to promote its synthesis such as Toll-like receptor activation (TLRs), prostaglandins, adipokines, stress response, and other cytokines (4). IL-6 can bind either the membrane bound IL-6 receptors (mIL-6R) or soluble IL-6 receptors (sIL-6R) (5). IL-6 family cytokines utilize gp130 for signal transduction through gp130 homodimers or GP130-containing heterodimers. While IL-6R is mainly expressed on immune cells and hepatocytes, gp130 is ubiquitous, which explains IL-6’s diverse roles in the body. In the classical pathway of signal transduction, IL-6 binds to the membrane bound IL-6R. Binding of IL-6- IL-6R complex to GP130 results in phosphorylation of JAK family kinases that are constitutively associated with the cytoplasmic region of GP130. In the second pathway known as trans-signaling, IL-6 binds to soluble IL-6 receptor (sIL-6R) which is created by alternative mRNA splicing or is shed from cells after cleavage by ADAM17 (metalloprotease) (6). IL-6 complexed with sIl-6R then binds the GP130. Thus, trans-signaling pathway allows for the activation of cells that do not express the IL-6R on their membranes (7). A third pathway of IL-6 signal transduction was recently described as ‘trans- presentation.’ This pathway is specific to dendritic cells that present IL- 6-mIL-6 complex to T cells expressing gp130 and primes them to become pro-inflammatory Th17 subsets (8).

## 2 Homeostatic role of IL-6 in health and infection

IL-6 secretion is stimulated during inflammatory response secondary to tissue injury or infection. After it is produced, it moves through the blood stream to the liver, triggering production of acute phase reactants such as C-reactive protein (CRP), serum amyloid A (SAA), and α1-antichymotrypsin, fibrinogen and haptoglobin (9). One of the effects of IL-6 is stimulation of hepcidin production, which blocks iron transportation from the gut. When this pathway is activated chronically, it causes what we know as anemia of chronic disease. IL-6 also increases zinc importer (ZIP14) expression on hepatocytes, inducing hypozincemia seen in inflammation, and thus delaying wound healing, among other effects of low zinc on the immune system (10). Once IL-6 reaches the bone marrow, it increases maturation of megakaryocytes, thus increasing the number of platelets, explaining why thrombocytosis is often seen during inflammatory response. Together with tumor necrosis factor alfa (TNF α) and IL-1, IL-6 is an important pyrogenic cytokine affecting lymphocyte trafficking (11). In mouse and rabit models, intravenous or intracerebroventricular introduction of IL-6 leads to increased body temperature (12). At the time of pyrogenic response, IL-6 trans-signaling aids in the multistep adhesion cascade promoting the entry of blood-borne lymphocytes across ‘gate-keeper’ high endothelial venules (HEVs) in lymph nodes and Peyer patches. In this context, primary tethering and rolling of lymphocytes along the HEVs as well as during secondary firm arrest of lymphocytes in HEVs, before they can migrate into the surrounding parenchyma, is potentiated by IL-6 trans-signaling. This sequence of events increases the probability that patrolling lymphocytes with encounter the sequestered antigens within the lymphoid organs. This illustrates how IL-6 sets up a framework of how pyrogenic response activates the lymphocyte–HEV–IL-6 trans-signaling biological axis to promotes immune surveillance. The cytokine helps control differentiation of monocytes into macrophages by regulating the expression of macrophage colony-stimulating factor (13). Macrophages are effectors cells of the innate immune response and one of the first line’s of defense against infections. They phagocytose bacteria and secrete antimicrobial proteins and pro-inflammatory cytokines to further potentiate the inflammatory response. Macrophages can present antigens to T cells. In addition, they play an important role in clearing the debris of the damaged or diseased cells through programmed cell death (14). IL-6 promotes Th2 response by inhibiting Th1 polarization (15). IL-6 induces CD4 T cells to secrete IL-4 that directs polarization to Th2. It also decreases IFNγ secretion by CD4 T lymphocytes, a cytokine critical for Th1 polarization. In Th1 cells, reduction of IFNγ leads to decreased T cell activation (16, 17). In conjunction with transforming growth factor beta, IL-6 induces CD4s to differentiate into Th17, a subset pathogenic in autoimmune mediated diseases but critical in the clearance of infectious agents from the mucosal sites (18). In addition, in synergism with IL-7 and IL-15, IL-6 empowers T cell differentiation and cytolytic ability (19). IL-6 is a growth factor for B cells (20), inducing their maturation and differentiation into plasma cells and increasing their survival (21). It stimulates B-cell IgG production by regulating the expression of IL-21 (22).

## 3 IL-6 in the central nervous system

In the central nervous system, IL-6 is generated within the cortical, brainstem, cerebellar and spinal cord areas. It is also secreted by the brain’s endothelial cells, modulating surrounding cell’s health and behavior (23). IL-6 is constitutively expressed at low levels by astrocytes (24, 25) and microglia (26) and, in certain scenarios such as injury, by neurons (27, 28). IL-6 effects are multifaceted and depend on the environment of the neuron and whether it is located in the central or peripheral nervous system, ranging from aiding in neurogenesis and neuro-regeneration after injury to promoting neurodegeneration and cell death. IL-1β, a pro-inflammatory cytokine secreted during infection or any CNS injury (as an example, traumatic brain injury, stroke, etc) also induces astrocytes and neurons to produce IL-6 (29, 30).

Astrocytes, neurons and microglia express the receptors for IL-6 (IL-6R) (31–33). In addition, given that gp130 is widely expressed in CNS tissue, IL-6 down-stream effects can take place via gp130-mediated trans-signaling (34). Convergence of IL-6 down-stream effects at JAK/STAT signaling pathway and inducing STAT3 phosphorylation enables pro-neuroregenerative effects of neurotrophins such as nerve growth factor in the peripheral sensory nerves (35). IL-6 acts as a neurotrophic factor for dopaminergic neurons in the midbrain and cholinergic neurons in the basal forebrain and septum (36, 37). It also influences neuronal excitability and helps regulate several voltage-gated and receptor-mediated channels (38).

Overall while IL-6 appears to be an important play in CNS health, its dysregulation leads to pathological effects. Increased levels of intrathecal IL-6 (albeit not the only cytokine elevated) have been found in various brain disease states ranging from traumatic brain injury, schizophrenia, depression (39), neuromyelitis optica spectrum disorder (40) to Alzheimer’s (41) and Lewy body dementia (LBD) (42). However, studies measuring IL-6 levels have produced inconsistent results due to limited ability to evaluate true CNS parenchymal levels of IL-6, including the timing of such evaluation in relation to the acute injury. Indeed, quantifying interstitial IL-6 levels inside the brain in living subjects is not feasible, and most studies have relied on measuring cerebrospinal fluid level of IL-6. In animal studies of experimental CNS injury, the level in the interstitial fluid (measured via microdialysis probe implantation) has been found to be 10 fold higher than in the CSF (43). However, in one study of severe traumatic brain injury, human subjects had similar levels of IL-6 in both CNS parenchyma and CSF as measured by dialysate (44), an invasive brain technique reviewed by Stovell et al. (45). In disorders associated with elevated systemic levels of IL-6, decreased cognitive function has been observed in humans and reproduced in animal models. In patients with cerebral vascular disease, those with dementia had higher levels of serum IL-6 in comparison to those without cognitive sequela (46). Elevated serum IL-6 levels have been associated with poorer cognitive performance in healthy subjects (47, 48). IL-6 in the blood may gain access into the CNS via leaky blood brain barrier and may have a direct effect on the blood brain barrier permeability, as discussed in the paragraph below.

## 4 IL-6 and the blood brain barrier

Intact blood brain barrier is critical to the homeostatic maintenance of the central nervous system (CNS) compartment, regulating the bi- directional traffic of fluids and solutes between the peripheral blood and the CNS microenvironment. Disruption of this barrier is linked to a number of inflammatory and neurodegenerative conditions. The neuro-inflammatory cascade accompanying the disruption of BBB is strongly linked to the elevated levels of cytokines such as IL-6 and TNF-α, among others. In a murine model of ischemic brain injury, IL-6 was noted to help decrease BBB integrity (49). In the ovine fetus model of ischemic insult increasing blood brain barrier permeability, 24 hours after ischemia, blocking IL-6 with a monoclonal antibody infusion attenuated ischemia-related increases in BBB permeability and modulated tight junction and PLVAP (plasmalemma vesicle protein) expression in fetal brain (50). Another murine model of inflammation associated with atherosclerosis and its effects on BBB showed that IL-6 produced in microvessels contributes to BBB impairment (51). Within the context of neuromyelitis optica (NMOSD), *in vitro* and *ex vivo* BBB models demonstrated that blocking IL-6 suppressed the NMO-IgG-induced transmigration of T cells and barrier dysfunction. In the *in vivo* study, blocking IL-6 signaling suppressed the migration of T cells into the spinal cord and prevented the increased BBB permeability (9). Even in the absence of microbial invasion of the CNS compartment, systemic inflammation associated with increased leakiness of BB results in increased lymphocyte trafficking into the brain, increasing the influx of natural killer cells, neutrophils and macrophages (52). Decreased BBB permeability has been implicated in the pathogenesis of many infectious, autoimmune and neurodegenerative conditions, including NMOSD, MS, HIV-associated dementia complex, Alzheimer’s and others. The effects of IL-6 blockade thus may be pertinent to a number of autoimmune and neurodegenerative conditions, in which blood brain barrier is dysregulated.

## 5 IL-6 and autoimmunity

Dysregulation of IL-6 axis is known to be involved in the inflammatory pathways of several autoimmune disorders such as rheumatoid arthritis, Castleman’s syndrome, idiopathic juvenile arthritis, neuromyelitis optica spectrum disorder, autoimmune epilepsy and others. While discussion of the diverse immunological mechanisms of these disorders is beyond the scope of this review, IL-6 dysregulation appears to be an integral part of these processes. While expression of IL-6 is tightly regulated by transcriptional and post-transcriptional mechanisms, in situations where the synthesis of IL-6 is chronically elevated, the inflammatory cascades that ensue lead to the pathological effects of chronic inflammation and autoimmunity. These effects can be explained by the pleotropic effects of IL-6 on the innate and adaptive immune system. Together with transforming growth factor (TGF)-β, IL-6 induces differentiation of CD4 T cells into the pro- inflammatory Th17 subsets, implicated in the pathogenesis of many autoimmune conditions (53). Moreover, IL-6 reduces TGF-Beta induced CD4 differentiation into the T regulatory subset, thus decreasing immune system’s natural brakes on the inflammatory response (54). Dysregulation of the Th17/Treg balance leads to the loss of immune tolerance and increases risk of a number of autoimmune conditions and chronic inflammation (55). Furthermore, IL-6 increases T-follicular helper-cell differentiation and production of IL-21 responsible for increased level of IgG, including IgG4. It induces B cells to become plasma cells and secrete IgG, thus chronic inflammation is associated with hypergammaglobulinemia. IL-6 further induces differentiation of CD8+ T cells into cytolytic T cells (56). It increases vascular endothelial growth factor (VEGF) and promotes angiogenesis and vascular permeability (57). Within the CNS compartment, in response to local inflammation or injury, astrocytes and microglia secrete IL-6, promoting downstream demyelination and contributing to oligodendrocyte and axon damage, as seen in NMOSD (40).

## 6 IL-6 axis modulating therapeutics

First clinical application of murine anti IL-6 monoclonal antibody was trialed in a patient with multiple myeloma (MM); it improved tumor burden and suppressed inflammatory acute phase responses, but it led to accumulation of IL-6- antibody immune complexes in the blood, preventing elimination of IL-6 and creating high level of IL-6, highlighting the need for IL-6 receptor blockade instead (58). Later, a clinical trial failed to show improved outcomes of MM when anti IL-6 agent was added to the typical regimen (the bortezomib–melphalan–prednisone regimen) (59, 60). Further research created anti Il-6R antibody that was humanized and given the name tocilizumab (developed by Kishimito and Chugai Pharmaceutical Co). Tocilizumab, binds to mIL-6R and sIL-6R and inhibits IL-6 signaling by preventing IL-6 from binding to IL-6R. In the 90s, tocilizumab was first used in a patient with Castleman’s disease, a lymphoproliferative disorder with a range of inflammatory symptoms. In response to IL-6R blockade, the fevers went down, the levels of C reactive protein (CRP) decreased to zero and hemoglobin levels increased. Subsequently to that a phase II clinical trial including 30 patients showed effectiveness of tocilizumab in Castleman’s disease, normalizing all markers such as CRP, serum amyloid A, hemoglobin, albumin, IgG and cholesterol (61). Tocilizumab was approved in 2009 in Europe and in 2010 in the United States. The discovery of elevated IL-6 levels in the synovium of rheumatoid arthritis led clinical trial of anti-IL6R antibody tocilizumab, paving its use in other autoimmune conditions (62) (5) such as:

1. Giant Cell Arteritis (GCA)
2. Rheumatoid arthritis
3. Polyarticular Juvenile Idiopathic Arthritis (PJIA)
4. Systemic Juvenile Idiopathic Arthritis (SJIA)
5. Cytokine Release Syndrome (CRS)
6. Adults and pediatric patients 2 years of age and older with chimeric antigen receptor (CAR) T cell-induced severe or life- threatening cytokine release syndrome.
7. Off-label use in neuromyelitis optica spectrum disorder and others.

The therapeutic benefit of tocilizumab led to the development of several other antibodies to IL-6 such as sirukumab, olokizumab, sartralizumab and clazakizumab. In 2022, FDA approved tocilizumab for emergency use for the treatment of COVID-19 in hospitalized pediatric patients 2 to less than 18 years of age who are receiving systemic corticosteroids and require supplemental oxygen, non-invasive or invasive mechanical ventilation, or extracorporeal membrane oxygenation (ECMO).

Two main anti-IL6 agents utilized in the United States and their FDA and some off-label uses are listed below in **Table 1**.

**Table 1:** Anti IL-6 agents: FDA approved and off-label uses.

Agent	FDA approved indications	Not FDA approved uses (off label) or in clinical trials
Tocilizumab	Rheumatoid arthritis Giant Cell Arteritis (GCA) Polyarticular Juvenile Idiopathic Arthritis (PJIA) Systemic Juvenile Idiopathic Arthritis (SJIA) Cytokine Release Syndrome (CRS) Adults and pediatric patients 2 years of age and older with chimeric antigen receptor (CAR) T cell-induced severe or life- threatening cytokine release syndrome.	MOGAD Autoimmune encephalitis Autoimmune epilepsy NMOSD NPSLE Sarcoidosis Behcet’s Adult-onset Still disease Ankylosing spondylitis Antibody-mediated rejection in renal transplantation Cardiac transplant rejection Crohn’s disease Hemophagocytic lymphohistiocytosis Polymyalgia rheumatica Psoriatic arthritis Relapsing polychondritis Retinal vasculitis Sjogren’s syndrome myelopathy Systemic lupus erythematosus Systemic sclerosis (excludes systemic sclerosis-associated interstitial lung disease) Systemic sclerosis-associated myopathy/polyarthritis Systemic vasculitis TAFRO syndrome Takayasu arteritis Thyroid eye disease Tumor nerosis factor receptor associated periodic syndrome (TRAPS) Uveitis
Sartralizumab	NMOSD (aquaporin 4 antibody positive)	MOGAD AE

## 7 IL-6 modulation in neuromyelitis optica

Neuromyelitis optica (NMOSD) is a rare relapsing autoimmune condition affecting central nervous system, pathogenically driven by anti-aquaporin 4 antibody activating terminal complement cascade with a resultant astrocyte damage and secondary demyelination. Patients affected by neuromyelitis optica experience attacks of longitudinally extensive transverse myelitis, unilateral or bilateral optic neuritis, among other disabling manifestations, that in some cases may be life-threatening (63) In this disease process, IL-6 appears to be instrumental by promoting plasmablast survival, increasing AQP4-IgG levels, enhancing pro-inflammatory T lymphocyte activation and impairing blood brain barrier (BBB). Moreover, levels of IL-6 have been noted to be increased in the serum and CSF, particularly during the attacks (40). In clinical trials, blocking IL-6 receptor, with a humanized monoclonal antibody tocilizumab resulted in significant reduction of relapses due to NMO. Tocilizumab went through several trials in neuromyelitis optica with positive results. In TANGO, an open- label, multi-centre, randomised, phase 2 trial recruited 118 adult patients (aged ≥18 years) with highly relapsing NMOSD who had a history of at least two clinical relapses during the previous 12 months or three relapses during the previous 24 months with at least one relapse within the previous 12 months. The patients were randomized into azathioprine vs tocilizumab groups. Fifty (89%) of 56 patients in the tocilizumab group were relapse-free compared with 29 (56%) of 52 patients in the azathioprine group at the end of the study (HR 0·188 [95% CI 0·076-0·463]; p&lt;0·0001); the median time to first relapse was also longer in the tocilizumab group than the azathioprine group (67·2 weeks [IQR 47·9-77·9] vs 38·0 [23·6- 64·9]; p<0·0001) (64). A recent meta-analysis evaluating safety and efficacy of anti IL-6 agents in NMOSD included a total of nine studies with 202 patients and found that a good proportion (76.95% CI: 0.61-0.91; p &lt; 0.001) of tocilizumab treated patients were relapse free at follow up. It also significantly reduced mean ARR (mean difference: -2.6, 95% CI: - 2.71 to - 1.68; p< 0.001) and but did not show significant difference in change in EDSS score (mean difference = - 0.79, 95% CI: - 1.89 to - 0.31; p = 0.16). Sakura trials of sartralizumab demonstrated significant reduction of the risk of relapses (65). In Sakura Star trial, 64 AQP4-IgG + adults were randomized to sartralizumab (n=41) or placebo (n=23). 77% of sartralizumab-treated patients were relapse-free at 96 weeks, compared to 41% placebo-treated patients, a 74% relative risk reduction (66). In Sakura Sky trial, 52 AQP4- antibody + adults taking certain immunosuppressant therapies were randomized to sartralizumab (n=26) or placebo (n=26). 91% of sartralizumab - treated patients were relapse-free at 96 weeks, compared to 57% placebo-treated patients, with a 78% relative risk reduction (67).

## 8 IL-6 modulation in myelin oligodendrocyte glycoprotein associated demyelination

Myelin oligodendrocyte glycoprotein associated demyelination (MOGAD) is another autoimmune demyelinating condition in which patients experience relapses of brain/optic nerve/spinal cord inflammation, similar to AQP4 positive NMOSD. The pathophysiology of MOGAD displays both antibody and complement-mediated CNS injury and includes elevated levels of IL-6 (68). A retrospective multicenter study evaluated the long-term safety and efficacy of tocilizumab (TCZ), a humanized anti-interleukin-6 receptor antibody in myelin oligodendrocyte glycoprotein-IgG-associated disease (MOGAD) and provided Class III evidence that long-term TCZ therapy is safe and reduces relapse probability in MOGAD. Fourteen MOGAD patients received TCZ for a median of 23.8 months (range 13.0-51.1 months), with an IV dose of median dose 8.0 mg/kg (range 6-12 mg/kg) monthly. The median ARR decreased from 1.75 (range 0.5-5) to 0 (range 0-0.9; p = 0.0011) under tocilizumab (69). Currently a Phase III, randomized, double-blind, placebo-controlled, multicenter trial evaluating the efficacy, safety, pharmacokinetics, and pharmacodynamics of satralizumab as monotherapy or in additional to baseline therapy in patients with MOGAD is ongoing (NCT05271409).

## 9 IL-6 modulation in Neurobehcet’s

Bechet’s disease (BD) is a relapsing, multi-system inflammatory vasculitis that can present with a remarkable heterogeneity in different patients, ranging from ocular, genital, skin to gastrointestinal to neurological involvements. Neurobehchet’s can present with parenchymal brain or spinal cord syndrome, peripheral nervous system involvement or venous sinus thrombosis. The etiopathogenesis of this disease remains poorly defined; however, both neutrophils and pathological activation of JAK/STAT pathway associated with IFNGR1 polymorphysms as well as the dysregulated inflammatory cytokine milieu with increases in IL-6 and IL-17 that promoted Th1/Th17 polarization have been implicated. Therapeutic agents used in the treatment of Behcet’s range from colchicine, azathioprine, mycophenolate mofetil, rituximab and include anti IL-6 agents. A systematic literature review of tocilizumab administration for the treatment of Behcet’s disease found 47 patients with a refractory disease in response to prior conventional and biologic agents with the mean disease duration was 99.5 ± 61.4 months. Tocilizumab was found to be effective in an organ-dependent fashion as an alternative treatment for refractory vasculo-, neuro-, oculo-Behcet’s disease, and secondary amyloidosis, but in mucocutaneous or join involvement (70). A multi-center study of BD patients treated with tocilizumab, refractory to standard treatment, studied 16 patients (10 men/6 women) found that tocilizimab is effective in BD with major clinical involvement. However, it did not seem to be effective in oral/genital ulcers or skin lesions (71).

## 10 IL-6 modulation in autoimmune encephalitis

Autoimmune encephalitis (AE) is an umbrella term for a diverse number of autoimmune conditions affecting the brain, with some being antibody mediated (targeting surface receptors: LGI1, NMDA, GlyR as an example), and some occurring due to a T cell mediated autoimmune response to an intracellular antigen (examples: Hu, Yo, KLCH 11). In a substantial number of cases, diagnostic workup does not return a specific antibody associated with a given case of autoimmune encephalitis. These cases are deemed to be seronegative, which means that the causative antibody is either not yet known or it is a T cell-mediated process. Treating these neurological syndromes is particularly challenging, as the underlying pathophysiology is poorly understood and could be either B or T cell-mediated. In these instances, after administering first line medications such as intravenous steroids, intravenous immunoglobulins or plasma exchange, many clinicians favor anti-proliferative agents targeting both B and T cells, such as mycophenolate mofetil or azathioprine, particularly if full improvement is not attained or there is a relapsing course (72). However, these medications often take many months to become fully effective. Because IL-6 results in B cell proliferation and increased antibody synthesis, increased polarization of T cells into Th17 pro-inflammatory subsets, decreases T regulatory cell ratios and induces proliferation of CD8 + cytotoxic T cells, blocking this pathway seems strongly appealing to target in cases of AE where the underlying pathophysiology is not fully elucidated. Moreover, anti IL-6 agents can be used in conjunction with mycophenolate mofetil and azathioprine in a relatively safe way, as seen in NMOSD trial (SakuraSky). Given that IL-6 has been shown to be elevated in the CSF of many subtypes of AE (73), it has been therapeutically trialed in several types of autoimmune encephalitis. In a large institutional cohort of patients with refractory to rituximab AE, the tocilizumab group showed more frequent favorable mRS scores at 2 months from treatment initiation and at the last follow-up compared with the other groups. 89.5% of the patients with clinical improvement at 1 month from tocilizumab treatment maintained a long-term favorable clinical response (74). Tocilizumab may be a good treatment strategy for treating AE refractory to conventional immunotherapies and rituximab. Currently A Phase III, Randomized, Double-Blind, Placebo-Controlled, Multicenter Basket Study To Evaluate The Efficacy, Safety, Pharmacokinetics, And Pharmacodynamics Of Sartralizumab In Patients With Anti-N-Methyl-D-Aspartic Acid Receptor (NMDAR) Or Anti-Leucine-Rich Glioma-Inactivated 1 (LGI1) Encephalitis is ongoing (NCT05503264).

## 11 IL-6 modulation in autoimmune epilepsy

The association of seizures and CNS autoimmunity is well described. Not only have seizures been seen as a common manifestation in autoimmune encephalitis, but administrative database research has shown patients with systemic autoimmune disorders to be at increased risk of seizures as well (75). While the term ‘autoimmune epilepsy’ was initially suggested as a concept in 2002, the term has continued to gain popularity and use as further studies and cohorts of patients with intractable seizures often related to autoimmune encephalitis (76–78). In the recent International League Against Epilepsy (ILAE) Definitions and Classifications guideline, the category of “immune etiology” was introduced and further defined by the ILAE Autoimmunity and Inflammation Taskforce in 2020 (79, 80). When looking specifically at the proinflammatory cytokine IL-6, we know that IL-6 along with other pro- inflammatory cytokines such interleukin-1β (IL-Iβ) and IL-2, are typically concentrated in low quantities within the brain, but increase after seizures (81, 82). However, some of the best data between the involvement of IL-6 and seizures has been seen in patients with new-onset refractory status epilepticus (NORSE). NORSE is defined as refractory status epilepticus (RSE) that occurs in adults or children without active epilepsy and without a clear acute or active structural, toxic, or metabolic cause identified in the first few days (83). While the exact pathophysiological mechanisms underlying NORSE remains elusive, arguments often suggest that NORSE results from a post-infectious process leading to exacerbated cerebral inflammation. This is supported by frequent abnormal cerebrospinal fluid (CSF) with mild pleocytosis and mildly elevated protein levels (84–87). Additionally, there is a subtype of NORSE, febrile infection-related epilepsy syndrome (FIRES), where status epilepticus is preceded by febrile illness. In these patients polymorphisms in cytokine-related genes were found (88, 89) further eluding to this association. There have also been several studies reporting increases in the actual serum and/or CSF cytokine levels in patients with NORSE. In these patients, in addition to elevated IL-6, there have been Th1- associated cytokines/chemokines and other proinflammatory cytokines IL-1ß, and CXCL8 elevated in the CSF compared with patients with chronic epilepsy (89–94). The last of these studies in 2023, demonstrated a significant increase in the serum and CSF of IL-6 along with TNF-α, CXCL8/IL-8, CCL2, MIP-1α, and IL-12p70 pro-inflammatory cytokines/chemokines in patients with status epilepticus (SE) compared with patients without SE. Interestingly, NORSE patients with elevated innate immunity serum and CSF cytokine/chemokine levels had worse outcomes at discharge and at several months after the status epilepticus ended (93). Moreover, in immunotherapy with intrathecal dexamethasone or anakinra (anti IL-1) therapies, a subsequent decrease in CSF pro-inflammatory cytokine levels was found to be associated with clinical improvement (95, 96). Another recent study, looked at 6 patients with anti-NMDAR encephalitis NORSE and 5 with cryptogenic NORSE, and found CSF IL-6 and CXCL8 levels to be associated with an up- proteomic score and that has now been suggested as a promising indicator for assessment of the severity of NORSE (97). Besides, NORSE, temporal lobe epilepsy has also been demonstrated to have increased serum concentrations of IL-6 compared with those in healthy controls (98). Additionally, increased circulatory concentrations of IL-6 have been associated with high glutamic acid decarboxylase (GAD) antibody titers in patients with epilepsy (99). Overall, further research is needed to better understand the exact role IL-6 plays in seizure generation and epileptogenesis.

## 12 IL-6 modulation in neuropsychiatric lupus erythematosus

Systemic lupus erythematosus (SLE) is an autoimmune disease involving multiple organ systems and affecting about 1.5 million people in the United States. Neuropsychiatric lupus is an umbrella term for the etiologically diverse neurological manifestations associated with SLE. Cognitive dysfunction is a significant problem in patients with SLE. Up to 48% of patients with SLE perform poorly on MOCA test with evidence of cognitive impairment (100). Patients with SLE can present with various neurocognitive syndromes ranging from chronic cognitive changes, acute psychosis to acute confusional state (encephalopathy). One study found that in NPSLE patients (30.5 ± 11.5 years old) the median IL-6 levels in the CSF were 32 pg/ml as compared to IL-6 level of 3 pg/ml (median) in SLE patients without neuropsychiatric manifestations (101). Elevated IL-6 cerebrospinal fluid levels have a strong association with psychosis and acute confusional state in patients with SLE (102, 103). In fact, neuro-filament light chain (NFL) levels have been found to be positively associated with IL-6 levels, highlighting the interface of inflammatory cascade driving neuronal damage. (104) Moreover, recent studies showed that SLE is associated with the breakdown of the blood brain barrier, gray matter loss and cognitive impairment. Those patients with extensive BBB leakage were found to have lower global cognitive score with the presence of impairment on one or more cognitive tasks (105). Hirohata and colleagues have recently demonstrated that the breakdown of BBB in patients with SLE plays a critical role in the development of diffuse psychiatric/neuropsychological manifestations, due to allowing influx of anti-neuronal antibodies from systemic circulation into the brain. Paired serum and cerebrospinal fluid (CSF) samples were obtained from 101 SLE patients when they presented active neuropsychiatric manifestations (69 patients with diffuse psychiatric/neuropsychological syndromes [diffuse NPSLE] and 32 patients with neurologic syndromes or peripheral nervous system involvement [focal NPSLE]) and from 22 non-SLE control patients with non-inflammatory neurological diseases. The levels of albumin and IL-6 in CSF and sera were measured by ELISA. The found that serum IL-6 and CSF IL-6 levels were significantly elevated in acute confusional state compared to other NPSLE manifestations (cognitive dysfunction, psychosis). They showed an increased albumin quotient between CSF and serum to highly blood brain barrier break-down and found the degree of albumin quotient to be higher in patients with acute confusional state versus other manifestations. Interestingly, serum IL-6 levels were significantly correlated with the albumin quotient, highlighting the relationship between IL-6 and BBB permeability (106). Currently there are no dedicated SLE treatments targeting acute confusional state or chronic neurocognitive changes associated with SLE. Clinical trials evaluating anti IL-6R agents in the acute confusional state and cognitive changes in association with SLE are critically needed.

## 13 IL-6 modulation in sarcoidosis

Sarcoidosis is a multi-systemic granulomatous disease, most commonly affecting the lungs (107), also affecting central and peripheral nervous system. The disease is associated with a dysregulation of the Th17/T regulatory cell ratio, detected in peripheral blood and bronchoalveolar lavage (108). This imbalance is reversed by immunosuppressive therapy. Because IL-6 plays a role in CD4 polarization into Th17 subset and decreases T regulatory cell differentiation, therapeutic agents blocking IL-6 axis appear to be a reasonable weapon. In fact, severe cases of progressive sarcoidosis have been associated with genetic variations in IL-6 coding gene (109). In Slovenian population a promotor polymorphism in the IL-6 gene was found to be a risk factor for sarcoidosis (110). In addition, a recent study of neurosarcoidosis showed elevated IL-6 levels in the CSF with levels 50 pg/ml being associated with a higher risk of relapse or progression (111). A recent case series of 4 patients with sarcoidosis, refractory to other treatments, tocilizumab has been found to be effective, allowing for successful steroid tapering (112). However, there are also several cases published of paradoxical sarcoidosis onset in patients treated with anti-IL-6 agents for other disorders (113, 114). Currently, a phase II clinical trial is evaluating efficacy and safety of sarilumab in patients with glucocorticoid-dependent sarcoidosis (NCT04008069).

## 14 Discussion

Since its discovery decades prior, interleukin 6 has proven to be functionally pleiotropic, and when dysregulated, to participate in a number of inflammatory cascades underlying pathophysiology of a range of autoimmune conditions. From its role in blood brain permeability, antibody production by B cells, Th17 subset differentiation and effects on regulatory B cells, IL-6 has been found to be a central cytokine interfacing with the normal innate and adaptive immune system and autoimmunity. Successful therapeutic modulation of IL-6 axis have led to the approval of multiple therapeutic agents, with several clinical trial ongoing at this time. Because anti IL-6 monoclonal antibodies can be combined with other immunosuppressive medications such as azathioprine and mycophenolate mofetil, their use may be of further interest in other neuro-inflammatory conditions that currently have no FDA approved treatments.

## Author contributions

EG: Conceptualization, Funding acquisition, Writing – original draft, Writing – review & editing. SV: Writing – original draft.
